# Patient safety problems in community-based mental health services: A qualitative exploration

**DOI:** 10.1192/j.eurpsy.2022.1605

**Published:** 2022-09-01

**Authors:** P. Averill, N. Sevdalis, C. Henderson

**Affiliations:** King’s College London, Centre For Implementation Science, Institute Of Psychiatry, Psychology And Neuroscience, London, United Kingdom

**Keywords:** Qualitative research, Patient safety, Health services research

## Abstract

**Introduction:**

Existing research has seldom examined patient safety problems experienced by service users accessing community mental healthcare, with the growing evidence base focusing largely on safety in psychiatric inpatient settings. Accordingly, there is poor understanding of safety issues in community-based mental health services as perceived by service users, carers, and healthcare professionals.

**Objectives:**

This study aims to explore safety problems in adult community-based mental health services, their causation, and priority areas for improving the safety of care provided in these services.

**Methods:**

In-depth, semi-structured interviews and focus groups were conducted with users of community-based mental health services, carers, and healthcare professionals employed within these settings. Interview topic guides were designed jointly with stakeholders from these groups (N=7) and piloted (N=3). Interviews and focus groups will be transcribed, coded, and analysed using an inductive thematic analysis approach. Illustrative quotes will be extracted and used to describe the key themes that emerge from the analysis and their inter-relationships.

**Results:**

This presentation will provide an outline of patient safety as understood and experienced by key stakeholder groups. Study findings will explicate safety issues, healthcare system factors underpinning their causation, as well as practices which could improve safety in this context.

**Conclusions:**

This research will help to advance understanding of the nature of patient safety problems in community-based mental healthcare services for adults, based on the experiences of service users, carers, and healthcare professionals within these services. The research will address key evidence gaps and represents an important step towards identifying areas which warrant intervention to improve patient safety.

**Disclosure:**

NS is the director of London Safety and Training Solutions Ltd, which offers training in patient safety, implementation solutions and human factors to healthcare organisations and the pharmaceutical industry. The other authors have no competing interests.

